# Supplementary figures and images for: Three-dimensional culture of chicken primordial germ cells (cPGCs) in defined media containing the functional polymer FP003
Source: PLoS One. 2018 Sep 21;13(9):e0200515. doi: 10.1371/journal.pone.0200515 (PMC6150485; doi:10.1371/journal.pone.0200515)

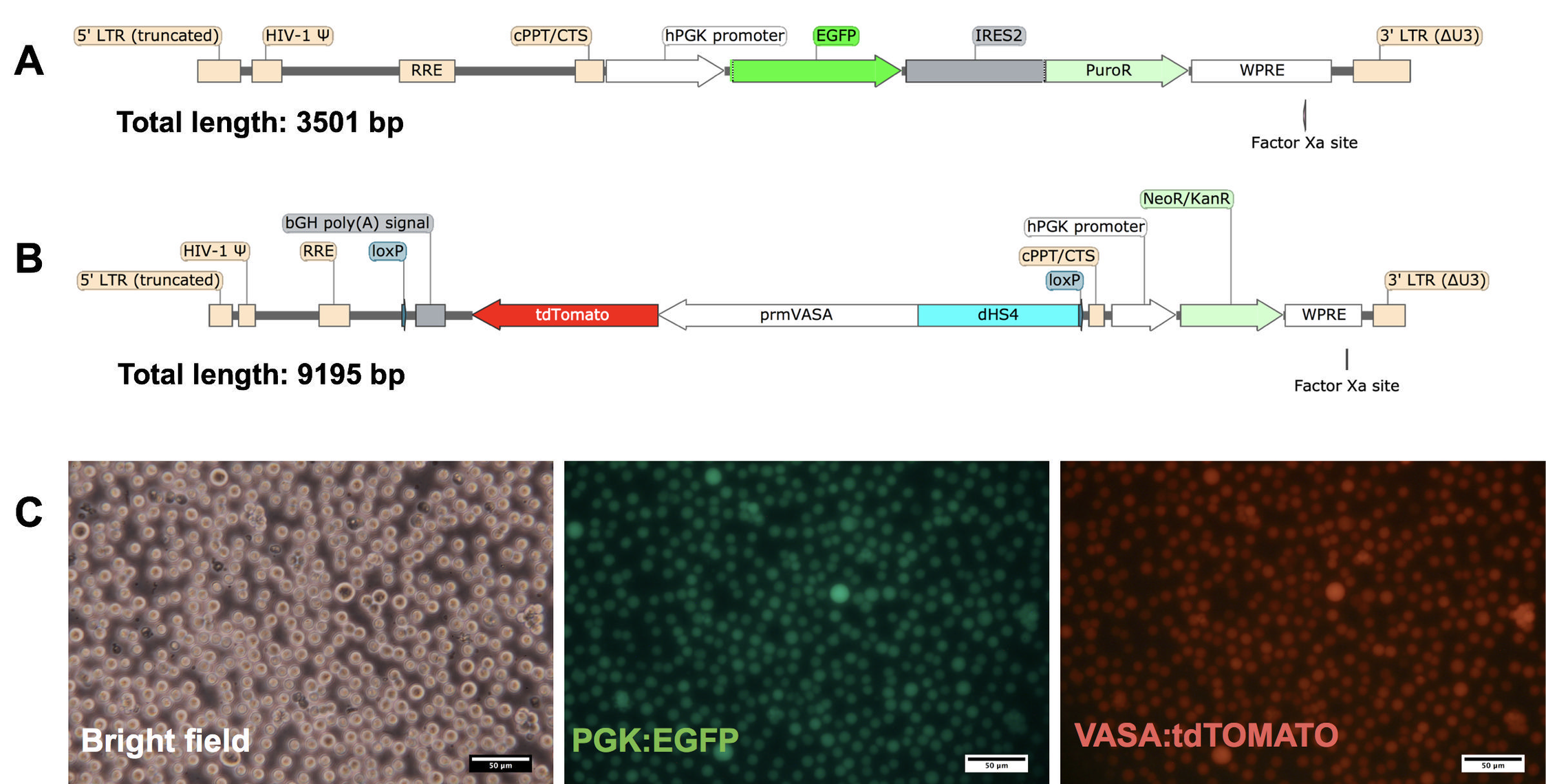

Supplement: S1 Fig — (A) Diagram of the cassette containing PGK:EGFP and associated plasmid features in the lentiviral vector. The total length of the fragment is 3501 bp. (B) Diagram of the cassette containing VASA:tdTOMATO and related plasmid features. The total length of the fragment is 9195 bp. (C) Images of cPGCs expressing these fluorescent reporters. Scale bar: 50 μm. (TIF) [file pone.0200515.s001.tif]

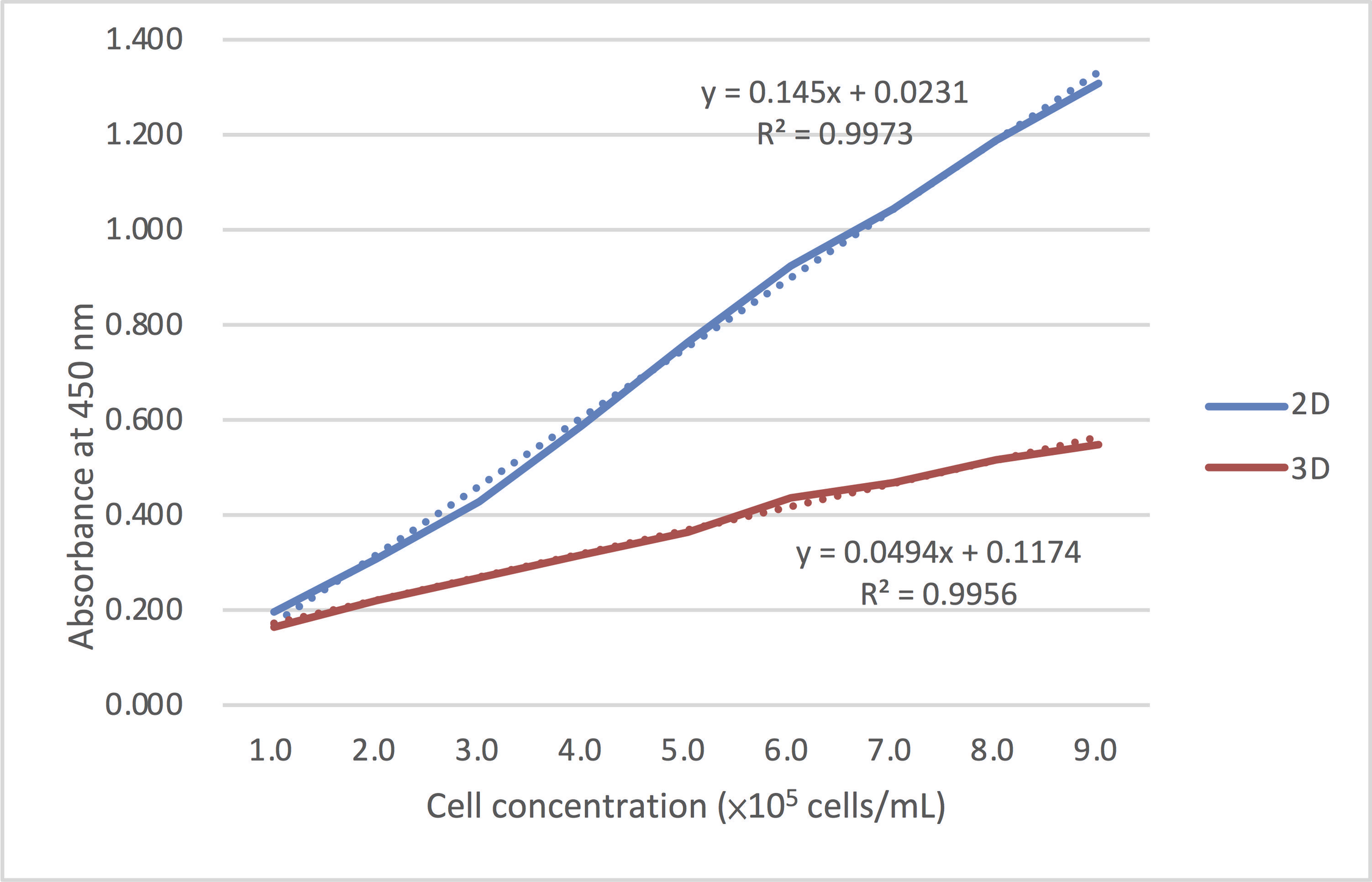

Supplement: S2 Fig — As determined by the CCK-8 assay, against the seeding density of cPGCs cultured in 2D or 3D medium. The formula and R-square value are provided next to each curve. Data are the mean. Each curve was generated using three replications. (TIF) [file pone.0200515.s002.tif]

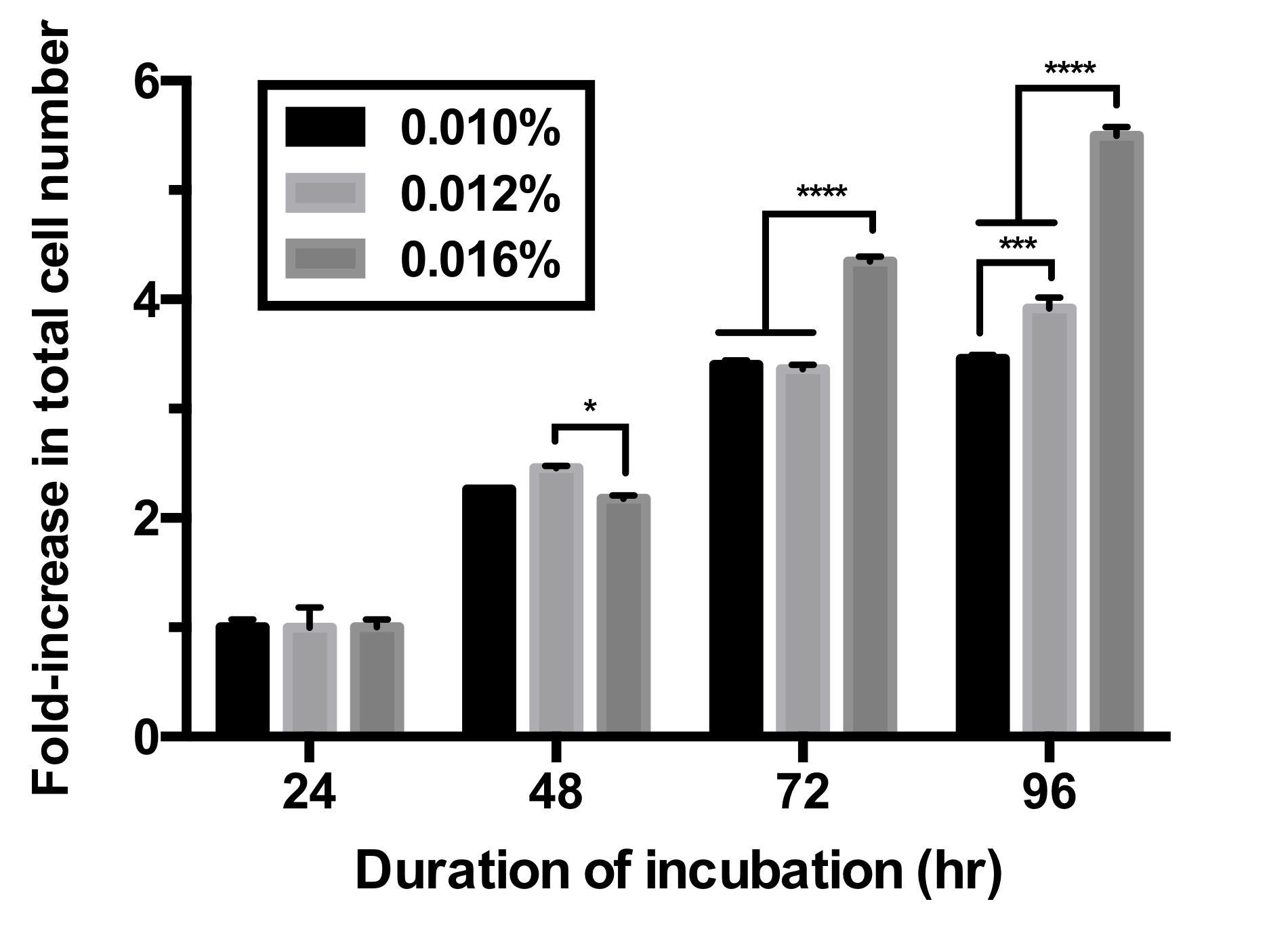

Supplement: S3 Fig — All data are mean ± SEM. * p < 0.05; *** p < 0.001; **** p < 0.0001 (TIF) [file pone.0200515.s004.tif]

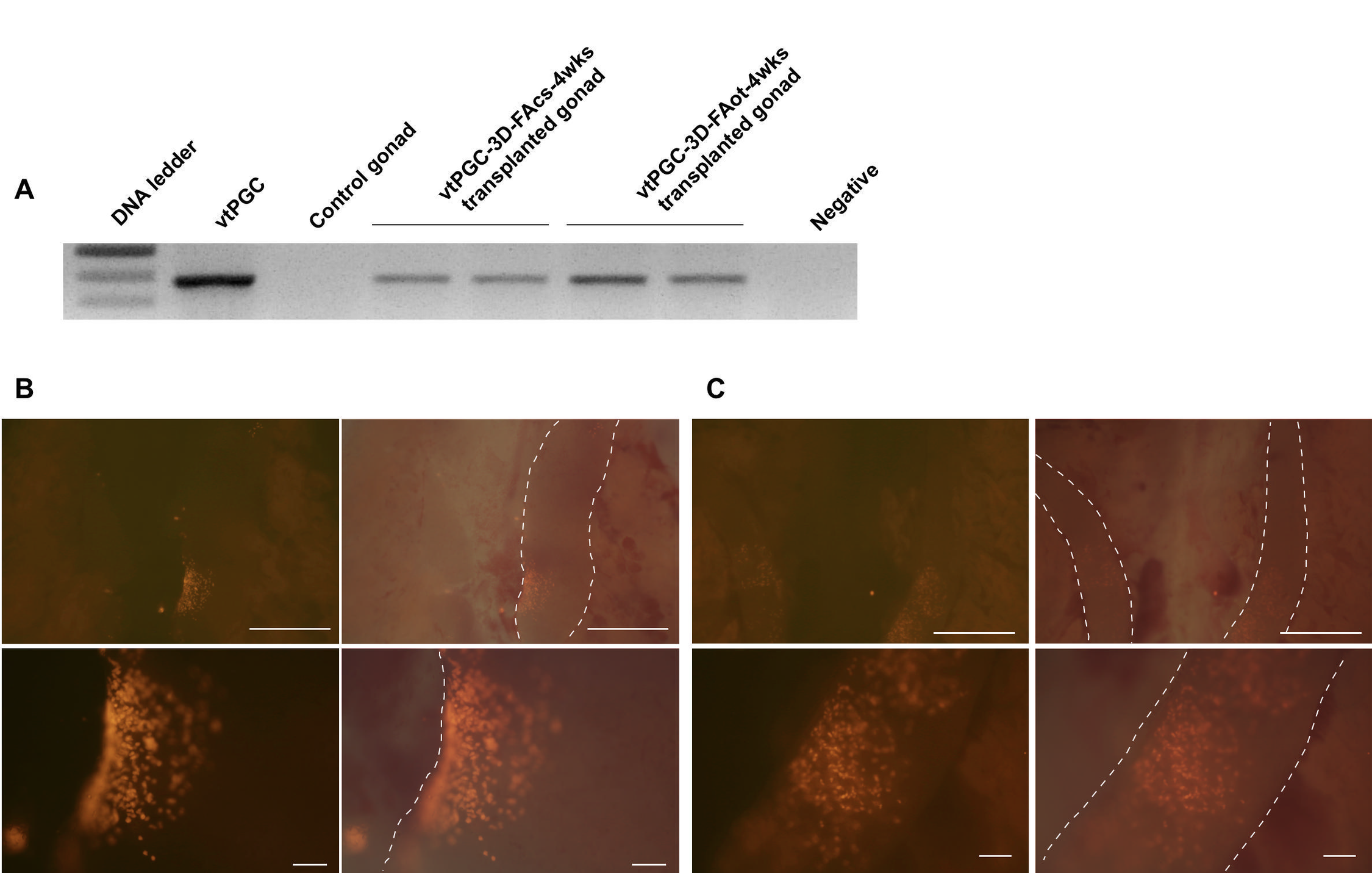

Supplement: S4 Fig — (A) The detection of tdTomato gene fragment in chicken embryonic gonads with or without the transplantation of 3D cultured vtPGCs by the PCR for a specific template. The template sized 375-bp represented the positive PCR product of tdTomato gene. (B) After PGC transplantation at E3, photographs indicated the E10 embryonic gonad with the colonization of the exogenic vtPGCs undergone the 4-week-culture in 3D-FAcs or (C) 3D-FAot medium. Scale bar: 1 mm (upper); 0.1 mm (below). (TIF) [file pone.0200515.s005.tif]
