# Supplementary material for: Three-dimensional culture of chicken primordial germ cells (cPGCs) in defined media containing the functional polymer FP003
Source: PLoS One. 2018 Sep 21;13(9):e0200515. doi: 10.1371/journal.pone.0200515 (PMC6150485; doi:10.1371/journal.pone.0200515)
Supplement: S1 Table — (DOCX) [file pone.0200515.s003.docx]

**S1 Table. Primer sets used for RT-PCR.**

| Gene | Full name | Forward primer (5’-3’) | Reverse primer (5’-3’) | Product length (bp) | Accession number |
| --- | --- | --- | --- | --- | --- |
| *DDX4* | DEAD (Asp-Glu-Ala-Asp) box polypeptide 4 (DDX4) | CAG ACC GCA TGC TTG ATA TG | CAG CCA GCC TCT GAA CTT CT | 135 | XM_013099917 |
| *DAZL* | Deleted in azoospermia-like (DAZL) | TCA CTG ACA GGA CTG GTG TTT C | ATT GCT GGT CCC AGT TTC AG | 127 | XM_013108529 |
| *POUV* | POU domain class 5 transcription factor 3 (POU5F3) | GTT GTC CGG GTC TGG TTC T | GTG GAA AGG TGG CAT GTA GAC | 189 | NM_001309372 |
| *NANOG* | Homeobox protein NANOG (NANOG) | GGT TTC AGA ACC AAC GGA TG | GTG GGG GTC ATA TCC AGG TA | 121 | XM_013092900 |
| *PRDM1* | PR/SET domain 1 (PRDM1) | CCC ACG AGT GTC AGG TTT GT | AGG TGC ACA AAC TGG GTG AA | 133 | XM_015284539 |
| *PRDM14* | PR/SET domain 14 (PRDM14) | AAG GCA AAG TGG TCA ACA CC | AGT TCA CCA GGG ACA TCC AG | 138 | XM_013101163 |
| *GAPDH* | Glyceraldehyde-3-phosphate dehydrogenase (GAPDH) | GAG GGT AGT GAA GGC TGC TG | CAT CAA AGG TGG AGG AAT GG | 113 | XM_005016745 |
| *tdTOMATO* | tdTomato fluorescent protein | GAG GTG ATG TCC AGC TTG GT | CAT CCC CGA TTA CAA GAA GC | 375 | N. A.^*^ |

***This primer set was designed based on the sequence of tdTOMATO.
